# Vegetable consumption among adults in Germany

**DOI:** 10.17886/RKI-GBE-2017-042

**Published:** 2017-06-14

**Authors:** Gert B. M. Mensink, Anja Schienkiewitz, Cornelia Lange

**Affiliations:** Robert Koch Institute, Department for Epidemiology and Health Monitoring, Berlin, Germany

**Keywords:** VEGETABLES, ADULTS, SURVEY, HEALTH MONITORING, GERMANY

## Abstract

Vegetables are part of a healthy diet and can help prevent various chronic diseases. According to the GEDA 2014/2015-EHIS study, 40.4% of women and 23.9% of men eat vegetables on a daily basis. The proportion of women who eat vegetables every day increases with age: from 31.9% of 18- to 29-year-olds to 48.3% of women aged 65 and above. Around one fifth of men under the age of 65 eat vegetables daily; this increases to 35.9% of men aged 65 or above. Across all age groups, women with higher levels of education are more likely to eat vegetables on a daily basis; the same can only be said about men in the 45 to 64 age group. Finally, women and men living in Saxony are most likely to eat vegetables every day; however, the differences between the federal states are marginal.

## Introduction

Vegetables are an important source of vitamins, minerals, trace elements, phytochemicals and dietary fibre; they can be defined as the edible parts of what are usually annual plants. A wide variety of vegetables exist, including cabbage, leafy, sprout, fruit, and root vegetables as well as bulbs, tubers and pulses. Mushrooms are also often counted as vegetables. The wide range of biologically active substances that are found in vegetables contributes to the fact that vegetable-rich diets are associated with a number of health benefits. In addition to a high nutrient density, most vegetables also contain a high volume of water and are therefore relatively low in calories [1]. At the same time, people who follow a diet containing a high proportion of vegetables usually eat other, physiologically less beneficial foods less often. Finally, as vegetables have a relatively low energy density, yet still create a substantial feeling of satiety, diets containing a high percentage of vegetables can help prevent weight gain and thus enable people to avoid obesity [1, 2].

There is convincing evidence that diets that include a large proportion of fruit and vegetables can help protect against coronary heart disease, hypertension and stroke; in addition, they can also improve the condition of patients suffering from these illnesses [1, 3-5]. A high vegetable consumption can probably help prevent various types of cancer; however, there is a marginal association between this consumption and the overall risk of cancer [1, 6-9]. Nevertheless, diets that include a large share of fruit and vegetables are associated with a lower overall risk of mortality, in particular, due to a lower risk of cardiovascular mortality [8].

For some time now, this has been reflected in the implementation of various health-policy measures aimed at encouraging people to eat more vegetables and fruit. The ‘5 a day’ campaign, which recommends that people eat five portions of fruit and vegetables every day, is probably one of the most well-known. A portion of fruit or vegetables may occasionally be replaced by a smoothie or a glass of fruit or vegetable juice; however, the fruit or vegetable content of these drinks should be no less than 100%. A portion is defined as a handful of fruit or vegetables [10, 11].

**GEDA 2014/2015-EHIS**

**Data holder:** Robert Koch Institute

**Aims:** To provide reliable informa tion about the population’s health status, health-related behaviour and health care in Germany, with the possibility of a European comparison

**Method:** Questionnaires completed on paper or online

**Population:** People aged 18 years and above with permanent residency in Germany

**Sampling:** Registry office sample; randomly selected individuals from 301 communities in Germany were invited to participate

**Participants:** 24,016 people (13,144 women; 10,872 men)

**Response rate:** 26.9%

**Study period:** November 2014 - July 2015

**Data protection:** This study was undertaken in strict accordance with the data protection regulations set out in the German Federal Data Protection Act and was approved by the German Federal Commissioner for Data Protection and Freedom of Information. Participation in the study was voluntary. The participants were fully informed about the study’s aims and content, and about data protection. All participants provided written informed consent.

More information in German is available at www.geda-studie.de

## Indicator

Eating sufficient amounts of vegetables is crucial to achieving a healthy, balanced diet. Population representative estimates of fruit consumption as an indicator of a healthy diet are, therefore, highly relevant for health policy. The GEDA 2014/2015-EHIS study assessed vegetable intake using the question: ‘How often do you eat vegetables, including freshly-squeezed vegetable juices? Please do not include potatoes.’ The study accepted the following responses: ‘Once or more a day’ ‘4 to 6 times a week’, ‘1 to 3 times a week’, ‘Less than once a week’ or ‘Never’. For the analysis that follows, these answers were grouped into three categories: once or more a day, at least once a week, and less than once a week. The results are listed according to gender, age, level of education and federal state. Differences between these groups are interpreted as statistically significant if the respective confidence intervals do not overlap.

The following analyses are based on data from 23,937 participants aged 18 and over with valid information on vegetable intake (13,098 women and 10,839 men). The calculations were carried out using a weighting factor that corrects for deviations between the sample and the German population (as of 31 December 2014) with regard to gender, age, district type and education. District type reflects a particular area’s degree of urbanisation and accounts for the regional distribution found in Germany. The International Standard Classification of Education (ISCED) was used to improve the comparability of the information that respondents provided about their level of education [12]. A detailed description of the methodology used in the GEDA 2014/2015-EHIS study can be found in German Health Update: New data for Germany and Europe, which was published in Issue 1/2017 of the Journal of Health Monitoring.

## Results and discussion

The German Nutrition Society (DGE) recommends that people eat fruit and vegetables every day [10]. However, in Germany, many adults do not meet this recommendation. According to the GEDA 2014/2015-EHIS study, 40.4% of women and 23.9% of men eat vegetables daily. This means that almost twice as many women as men consume vegetables every day (Table 1 and Table 2). In the GEDA 2012 study, 52.5% of women and 35.8% of men reported that they ate vegetables on a day-to-day basis [13]. The substantial decline between the two studies was also observed for fruit consumption and could have partly been caused by the different survey modes that were employed (a self-administered questionnaire was used in 2014/2015, whereas a telephone interview was conducted in 2012) as well as changes that were made to the questionnaire and its associated response categories. During GEDA 2012, respondents were contacted by telephone and asked ‘How often do you eat vegetables? Please do not include potatoes’. If the respondents asked for more detail, they were told: ‘Vegetables refers to raw vegetables like salad, cucumber, tomato and cooked vegetables.’ The following answers were accepted: ‘Every day’, ‘At least once a week’, ‘Less than once a week’ and ‘Never’. In the case of the GEDA 2014/2015-EHIS study, the question was asked in writing (as explained above under ‘Indicator’). Importantly, the GEDA 2012 study may have resulted in a tendency among respondents to choose ‘Every day’, even though they only ate vegetables 5 or 6 times a week. The fact that response categories were specified in this way presumably partly explains why GEDA 2012 produced higher figures for daily intake than GEDA 2014/2015-EHIS.

**Figure 1:**
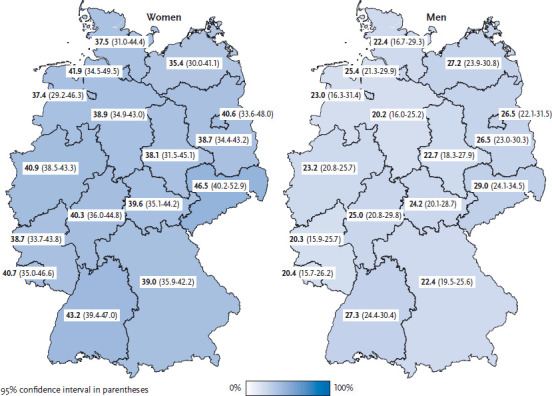
Daily intake of vegetables according to gender and German federal state (n=13,098 women; n=10,839 men) Source: GEDA 2014/2015-EHIS

Women tend to eat more vegetables on a daily basis with increasing age: in the 18-to-29 age group, 31.9% of women consume vegetables daily, with 48.3% doing so in the 65 and above age group (Table 1). About one fifth of men under the age of 65 eat vegetables every day. It is only from the age of 65 that 35.9% of men eat vegetables on a daily basis. This value is considerably higher than the figures observed among men of younger age groups (Table 2). Previous studies have also shown rising levels of vegetable intake with increasing age [13, 14]. In fact, daily vegetable intake is most common in the age group of 65 and older. This could be due to the fact that people in this age group are more concerned both with their health and following a healthy diet. Moreover, they are less likely to be employed and, therefore, have more time to choose, buy and prepare their own food. They also cook more often than younger people, preparing their own meals every day or almost every day [15]. Across all age groups, women with higher levels of education are significantly more likely to eat vegetables on a daily basis. Among men, the only significant difference linked to educational levels is found among 45- to 64-year-olds. For both genders together, vegetable intake in Saxony is significantly higher than the national average; however, for women and men separately there is no significant difference. No other significant differences were identified between the federal states (Figure 1).

Percentage shares of daily vegetable intake are markedly lower than for fruit. According to analyses of the German National Nutrition Survey II, in 2006, 86.3% of women and 88.5% of men did not meet the recommendations made by the German Nutrition Society (an intake of 400 g of vegetables per day, not including juices) [16]. According to the results of the German Health Interview and Examination Survey for Adults (DEGS1), women in Germany eat an average of 1.0 portions of vegetables per day; for men, the figure stands at 0.8 [17]. Therefore, an even greater increase in vegetable intake needs to be achieved in comparison to fruit intake, especially among men, young adults and people with lower levels of education.

## Key statements

- 40% of women and 24% of men in Germany eat vegetables every day.
- Daily vegetable intake among women is higher with age.
- Across all age groups, women with higher levels of education are more likely to eat vegetables on a daily basis.

## Figures and Tables

**Table 1:** Vegetable intake among women according to age and educational status (n=13,098) Source: GEDA 2014/2015-EHIS

Women	Once or more a day		At least once a week		Less than once a week	
	%	(95% CI)	%	(95% CI)	%	(95% CI)
**Women total**	**40.4**	**(39.3-41.5)**	**55.9**	**(54.8-57.1)**	**3.7**	**(3.3-4.1)**
**18-29 Years**	31.9	(29.6-34.3)	61.8	(59.3-64.2)	6.4	(5.2-7.8)
Low education	29.7	(23.8-36.3)	57.3	(50.8-63.6)	13.0	(9.3-17.9)
Medium education	29.7	(26.9-32.6)	65.2	(62.1-68.1)	5.2	(4.0-6.6)
High education	44.4	(39.2-49.6)	54.4	(49.2-59.6)	1.2	(0.6-2.5)
**30-44 Years**	38.2	(35.9-40.5)	57.8	(55.6-60.0)	4.0	(3.1-5.0)
Low education	29.6	(23.3-36.6)	60.7	(53.0-67.9)	9.7	(6.1-15.1)
Medium education	34.8	(31.9-37.8)	61.6	(58.7-64.4)	3.6	(2.7-4.8)
High education	51.2	(48.0-54.4)	47.1	(43.9-50.3)	1.6	(1.0-2.7)
**45-64 Years**	39.7	(38.0-41.4)	57.2	(55.5-58.9)	3.1	(2.6-3.7)
Low education	34.5	(30.4-38.8)	60.5	(56.0-64.7)	5.1	(3.5-7.4)
Medium education	37.0	(34.9-39.1)	60.1	(57.9-62.2)	3.0	(2.3-3.8)
High education	53.3	(50.4-56.3)	44.9	(41.9-47.9)	1.8	(1.1-2.7)
**≥ 65 Years**	48.3	(46.0-50.6)	49.1	(46.8-51.5)	2.6	(2.0-3.4)
Low education	47.2	(43.3-51.1)	49.8	(46.0-53.6)	3.1	(2.0-4.6)
Medium education	46.7	(43.5-50.1)	50.8	(47.5-54.2)	2.4	(1.6-3.6)
High education	60.9	(55.6-65.9)	37.6	(32.7-42.8)	1.5	(0.6-3.9)
**Total (women and men)**	**32.3**	**(31.5-33.2)**	**62.1**	**(61.3-62.9)**	**5.6**	**(5.2-6.0)**

CI=confidence interval

**Table 2:** Vegetable intake among men according to age and educational status (n=10,839) Source: GEDA 2014/2015-EHIS

Men	Once or more a day		At least once a week		Less than once a week	
	%	(95% CI)	%	(95% CI)	%	(95% CI)
**Men total**	**23.9**	**(22.9-25.0)**	**68.6**	**(67.4-69.7)**	**7.5**	**(6.8-8.3)**
**18-29 Years**	20.1	(17.9-22.6)	67.6	(64.8-70.2)	12.3	(10.3-14.6)
Low education	23.5	(18.5-29.4)	58.5	(52.2-64.6)	18.0	(13.1-24.1)
Medium education	18.0	(15.3-21.2)	70.5	(67.0-73.8)	11.5	(9.0-14.4)
High education	23.3	(18.5-29.0)	71.2	(65.3-76.4)	5.5	(3.3-9.1)
**30-44 Years**	19.1	(17.3-21.1)	72.4	(70.3-74.5)	8.5	(7.1-10.1)
Low education	18.6	(13.0-25.8)	68.9	(61.3-75.6)	12.6	(7.9-19.4)
Medium education	16.4	(13.9-19.2)	74.1	(71.0-77.0)	9.5	(7.6-11.8)
High education	24.7	(21.7-28.1)	70.7	(67.1-74.0)	4.6	(3.2-6.5)
**45-64 Years**	21.6	(20.2-23.0)	71.3	(69.6-72.9)	7.2	(6.3-8.1)
Low education	17.2	(13.8-21.2)	66.9	(62.2-71.2)	15.9	(12.6-19.9)
Medium education	19.3	(17.4-21.3)	73.2	(70.9-75.4)	7.5	(6.3-8.9)
High education	27.0	(24.8-29.4)	69.5	(67.0-71.9)	3.5	(2.7-4.5)
**≥ 65 Years**	35.9	(33.7-38.3)	60.8	(58.5-63.2)	3.2	(2.5-4.1)
Low education	35.2	(30.5-40.2)	60.7	(55.4-65.7)	4.1	(2.5-6.7)
Medium education	33.5	(30.1-37.0)	63.3	(59.7-66.7)	3.3	(2.3-4.5)
High education	40.9	(37.7-44.2)	56.3	(53.1-59.5)	2.8	(1.9-4.1)
**Total (women and men)**	**32.3**	**(31.5-33.2)**	**62.1**	**(61.3-62.9)**	**5.6**	**(5.2-6.0)**

CI=confidence interval
